# Performance evaluation of the properties of gel patches based on the cross-linking reaction of partially neutralized sodium polyacrylate

**DOI:** 10.1371/journal.pone.0342546

**Published:** 2026-04-28

**Authors:** Zhaoying Qi, Enzhao Wang, Yuzhou Cao, Rongshuang Tang, Jiquan Zhang, Jing Wu, Dawei Hao, Minchen Liu

**Affiliations:** 1 Innovation Research Institute of Traditional Chinese Medicine, Shanghai University of Traditional Chinese Medicine, Shanghai, China; 2 Engineering Research Center of Modern Preparation Technology of TCM of Ministry of Education, Shanghai University of Traditional Chinese Medicine, Shanghai, China; 3 School of Science, National University of Singapore, Singapore, Singapore; 4 School of Pharmacy, Zhejiang Pharmaceutical University, Ningbo, China; 5 Materials Science and Engineering, East China University of Science and Technology, Shanghai, China; National University of Rosario, ARGENTINA

## Abstract

Four types of partially neutralized sodium polyacrylate (PNSP) were prepared using two commonly used neutralization degrees of sodium acrylate/acrylic acid in molar ratios of 50/50 and 35/65. The performance and rheological properties of these four gel patches were analyzed through quality evaluation and rheological assessment. Concurrently, the structure and performance of the four gel patches were discussed based on electronic nose testing, cryo-scanning electron microscopy (cryo-SEM), and thermogravimetry-mass spectrometry coupling analysis (TGA-MS). The results indicated that no significant changes occurred in the quality or rheological properties of the four gel patches over a 30-day period. Electronic nose testing, cryo-SEM, and TGA-MS results indicated that PNSP with equivalent neutralization levels had no significant impact on the mass, formulation characteristics, or structure of the gel patches.

## 1. Introduction

**Fig 1:**
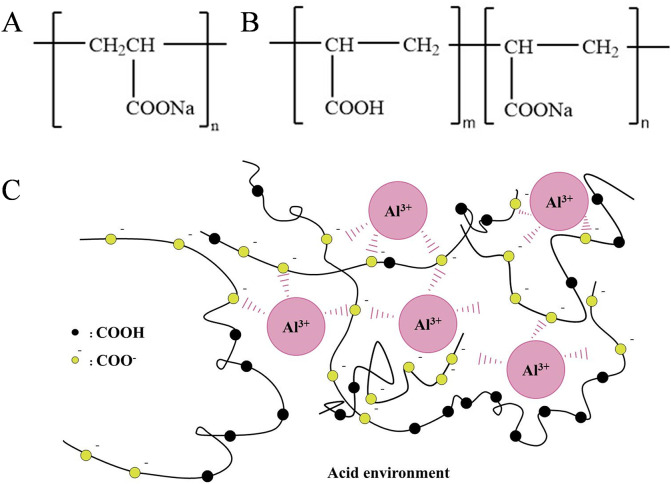
A: Structure of PNSP; B: The structure of sodium polyacrylate; C: Cross-linking of PNSP with Al^3+^.

Gel patches, as an important transdermal drug delivery system, are widely used [1,2]. Partially neutralized sodium polyacrylate (PNSP), owing to its excellent water-soluble, enhancing viscosity, and high molecular weight, acts as a small anion and has become one of the most commonly used matrix materials in gel patch formulations [3–5]. It serves as a key component in forming the cross-linked network structure. The structure of PNSP is shown in Fig 1A. Currently, this material can be used in pharmaceutical gel patch [6], fever cooling patches [7], medical appliances, and other products. The cross-linking mechanism of PNSP involves pre-dispersing it in moisturizers such as glycerin. Then, water is added, and raw material for hydrogel (gel state) is made by kneading with metal ions, like aluminum and organic acids, as crossing-linking agents (Fig 1C) [8], thereby imparting the required viscoelasticity and cohesion to the patch.

PNSP is a polymer of acrylic acid and sodium acrylate or a product of acrylic acid polymer partially neutralized by sodium hydroxide. Sodium polyacrylate [(C_3_H_3_NaO_2_)n], is a common water-soluble polymer, which is made from sodium salt and acrylic acid polymerizing in the presence of an initiator [9]. It has straight-chain polymers with hydrophobic and hydrophilic groups, which can flocculate into clusters when exposed to water. As a new type of functional polymer materials and important chemical products, the molecular chain of sodium polyacrylate contains a large number of carboxyl groups, with good hydrophilicity and polyelectrolyte properties. In addition, it also offers the advantages of non-toxicity [10,11], high adhesive strength [12], good stability [13], strong binding of cations [14] etc.; thus, it has a promising application prospect in the biomedical field [15–18]. The structure of sodium polyacrylate is shown in Fig 1B. Although sodium polyacrylate possesses numerous excellent properties, specific biomedical applications such as gel patches demand more refined material performance characteristics. Consequently, PNSP is frequently employed in gel patches. By controlling the monomer ratio of acrylic acid to sodium acrylic acid, PNSP retains the core advantages of sodium polyacrylate while adapting to different formulation requirements.

The number of donors bound to aluminum ions varies due to the different degrees of neutralization in PNSP, resulting in different material properties. These differences greatly affect the quality of gel patch preparation and the key properties of the gel patch. The characteristics of four types of partially neutralized sodium polyacrylate are shown in Table 1. However, there has been limited research on the effects of varying degrees of neutralization on the rheological properties, microstructure, and thermal stability of PNSP-based gel patches.

**Table 1:** Characteristics of PNSP of different types.

Types	neutralization degree	Viscosity (mPa·s/20 °C，0.2%)	pH (20 °C，0.2%)
	Sodium acrylate/Acrylic acid		
NP-700	50/50	500 ~ 650	6.2 ~ 6.8
AH-105X		500 ~ 650	6.0 ~ 7.0
NP-800	35/65	450 ~ 600	5.5 ~ 6.1
AH-106X		500 ~ 650	5.5 ~ 6.5

This study selected two representative PNSPs with neutral concentrations (50/50 and 35/65) to prepare gel patches, and examined the physical properties and consistency of the gel patches. Consistency was evaluated through quality assessment and electronic nose evaluation. Rheological properties over 30 days were analyzed using storage modulus, loss modulus, and loss tangent. Cryo-scanning electron microscopy (cryo-SEM) further revealed the internal structure of the gel patches, while thermogravimetric analysis (TGA) examined the thermal stability of PNSP and the gel patches. We aim to reveal the potential impact of PNSPs with varying degrees of neutralization on the performance of gel patches through comparative studies, thereby providing experimental evidence for the reliability and reproducibility of related scaffold materials under diverse application conditions.

## 2. Materials and instruments

### 2.1 Instruments

The following instruments were used in this study: vacuum kneading machine (ZNH-5L, Rugao Chenguang Kneading machine factory, China), hydrogel-coating machine (Suzhou Shunxinquan Machinery Co., Ltd, China), tensile testing machine (KJ-1065B, Guangdong Kejian Instrument Co., Ltd, China), electronic balance (OHAUS Instrument (Changzhou) Co., Ltd, China), electronic nose (PEN3, Airsense Co., Ltd, Germany), discovery hybrid rheometer (TA-20, TA Co., Ltd, USA), cryogenic high-resolution scanning electron microscopy (Gemini 300, Carl Zeiss AG, Germany), thermos gravimetric analyzer (TGA-550, TA Co., Ltd, USA), and scanning electron microscope (Quanta 2200, Thermo Fisher Scientific Inc., USA). Mass Spectrometry (MS, NETZSCH, Germany).

### 2.2 Materials

PNSPs (AH-105X, AH-106X, NP-700 and NP-800) were purchased from Toagosei Co., Ltd. (Tokyo, Japan) and Showa Denko KK (Kawasaki, Japan). Polyacrylic acid (AC-10H) was purchased from Toagosei Co., Ltd. (Tokyo, Japan). Sodium carboxymethylcellulose (CMC-Na) was purchased from Ashland Chemicals (Nanjing) Co. (Nanjing, China). Aluminum glycinate was purchased from Keiwa Kogyo Co., Ltd. (Takamatsu, Japan). Ethylene diamine tetraacetic acid (EDTA), tartaric acid, and potassium sorbate were purchased from Hunan Erkang Co., Ltd (Changsha, China). Glycerol was purchased from Zhejiang Suichang Huikang Pharmaceutical Co., Ltd. (Lishui, China). Titanium dioxide was purchased from Hunan Jiudian Hongyang Pharmaceutical Co., Ltd (Changsha, China). Medicinal polypropylene embossed film and non-woven fabric for gel patch preparation were purchased from Nantong Huakai New Material Technology Co., Ltd. (Nantong, China).

## 3. Methods

### 3.1 Preparation of gel patch [4]

**Fig 2:**
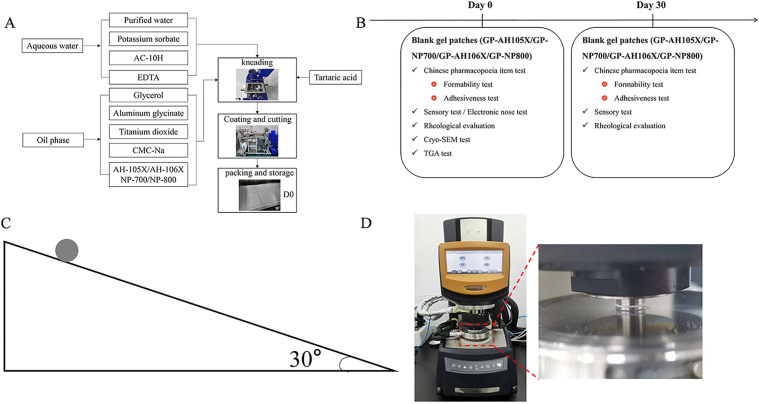
A: Preparation of the gel patch; B: The test items of gel patches on Day 0 and Day 30; C: Adhesiveness test device diagram; D: State of the sample on the rheometer.

The gel patch was prepared based on the existing literature (Fig 2A).

Using gel patches, the effect of two different models of PNSP (AH-105X and NP-700) with sodium acrylate/acrylic acid equal to 50/50 and two different models of PNSP (AH-106X and NP-800) with sodium acrylate/acrylic acid equal to 35/65 on the formation of the formulations was investigated in this study.

### 3.2 Establishment of evaluation indicator

Based on the above preparation procedure, we prepared gel patches using two different degrees of neutralization (sodium acrylate/acrylic acid equal to 50/50 and sodium acrylate/acrylic acid equal to 35/65) of PNSPs named GP-AH-105X, GP-NP-700, GP-AH-106X, and GP-NP-800, respectively. The degree of cross-linking of a gel patch changes over time since the cross-linking process typically involves chemical cross-linking reactions, where residual cross-linking agents continue to react with the reactive groups in the polymer chain over time [4]. Therefore, all prepared samples are stored at room temperature for several days to ensure successful cross-linking during testing. In this study, we took the time after 7 days of storage as day 0, and one month of storage on the basis of day 0 was recorded as day 30. The samples on day 0 and day 30 were tested, with Fig 2B showing the specific test items.

#### 3.2.1 Chinese pharmacopeia item test.

**3.2.1.1 Formability test:** Based on the method delineated in the Chinese Pharmacopoeia 2020 edition (General rule 0122) to measure the excipient properties of the gel patch, the gel patch surface should be free of flowing phenomenon.

**3.2.1.2 Adhesiveness test:** According to the ball slope stopping method delineated in the Chinese Pharmacopoeia 2020 edition (General rule 0952), the largest number of steel balls that the gel patch could stick to was recorded. A higher ball number was associated with a greater initial viscosity of the gel patch. The test setup diagram is shown in Fig 2C.

**3.2.1.3 Peel strength test:** According to the peel strength determination method delineated in the Chinese Pharmacopoeia 2020 edition (General rule 0952), record the load required to peel the polyester film from the surface of the gel patch. A higher load indicates a greater force required.

#### 3.2.2 Electronic nose test.

PNSP has been reported to be odorless; thus, we used an electronic nose to study the odor of the gel patch to comprehensively measure the odor of PNSP with different degrees of neutralization after making the preparations.

The brain’s ability to convert the mixtures of volatile organic compounds into recognizable odors is limited and varies from person to person, which explains the necessity of electronic nose. Like the human nose, the electronic nose analyzes odors [19–21]. Owing to its fast speed and reliable data, the electronic nose can scientifically and reliably evaluate the odor characteristics of gel patches. The PEN3 electronic nose sensor consists of 10 metal oxide semiconductor chemical sensing elements. Each of these elements has a different type of the main sensitive substance. Different gel patches present different odor-sensing signals on the sensor. The test method is as follows:

Samples (sampling day designated as Day 0) weighing 5 g each were placed in 50 ml centrifuge tubes, 1 tube for each sample volume (1 time in parallel). They were sealed with a sealing film, left 30 min for enrichment, and then tested. The direct headspace aspiration method was employed to insert the injection needle directly into a sealed centrifuge tube containing the sample. Thereafter, the electronic nose was used for determination. Measurement conditions were as follows: sampling time was 1 sec/group; sensor self-cleaning time was 100 sec; sample preparation time was 5 sec; the injection flow rate was 400 ml·min^-1^; analysis sampling time was 100 sec. Data were obtained from 68 to 70 seconds for summarizing and analyzing the results.

For sample differentiation analysis, the eigenvalues of 10 sensors were extracted and the raw data were analyzed using principal component analysis (PCA). When analyzing using PCA, it is possible to check the status of sample differentiation in each principal component. The loading plot can be used to determine which component contributes most significantly to the principal component.

#### 3.2.3 Rheological evaluation.

Rheology studies are important for gel patches. It can be used not only to assess the physical properties of gels [22–25], and also plays a critical role in the guidelines for semi-solid formulations [26–28]. Zhiyuan Hou et al’s [29] rheological analysis of the ketorolac tromethamine transdermal patch thoroughly revealed the rheological properties of the patch. At first, through amplitude scanning, the linear viscoelastic region was determined to be 10–1225 Pa, indicating that the patch can withstand daily skin pressure without deformation. Frequency scanning showed that G′ was consistently greater than G″, confirming the presence of a cross-linked network structure, irreversible deformation in the creep recovery experiment indicated that the material exhibited viscoelastic solid characteristics; and the rheological curve demonstrated a distinct shear thinning behavior. Based on multidimensional rheological data analysis and comprehensive consideration, the patch was found to be capable of both secure adhesion and flow spreading. In this study, we divided the gel patches into two groups based on the degree of neutralization of PNSP: a group of gel patches GP-AH-105X and GP-NP-700 made of PNSP with sodium acrylate/acrylic acid equal to 50/50 and a group of PNSP with sodium acrylate/acrylic acid equal to 35/65 made of gel platers GP-AH-106X and GP-NP-800. The rheological behavior of the two groups of gel patches was studied on days 0 and 30. The rheometer was employed to determine the rheological indexes of the gel patch and evaluate them to provide a scientific and reliable reference for the quality evaluation of the gel patch, including amplitude scanning, frequency scanning, and loss angle tangent.

**3.2.3.1 Amplitude scanning:** The linear viscoelastic region (LVR) of the sample was determined based on the amplitude scanning of the sample at a strain of 0.1% to 200% and an angular frequency of 10 rad/s. LVR is the region where the dynamic modulus does not change with strain. A larger LVR suggests a more stable internal structure, a higher deformation resistance, and a greater rigidity [30].

The gel patches were obtained and transferred to the sample tray of the rheometer (Fig 2D). Experimental parameters were set based on the actual production conditions with a temperature of 25 °C, angular frequency of 10 rad/s, and strain of 0.1% ~ 200%. The test was started when the temperature of the device rose to 25 °C.

**3.2.3.2 Frequency scanning:** Within the LVR, the strain was set to be 1%, and the angular frequency (ω) was 0.1 rad/s ~ 100 rad/s. Frequency scans of the samples were conducted to obtain the variation curves of elastic modulus (G′) and loss modulus (viscosity modulus, G″) at different frequencies and characterize the strength properties of the gel patch over time. G′ [31] reflects the energy stored in the hydrogel and elastic properties due to elastic deformation (reversible), and G″ [32] reflects the energy and viscous deformation (irreversible). The hydrogel is more elastic and mechanically stronger when the storage modulus of the hydrogel is greater than the loss modulus. It means that higher viscosity worsens mechanical strength [33,34].

**3.2.3.3 Loss angle tangent:** The loss angle tangent, also known as the loss factor, is the G″ to G′ ratio of a material. The loss factor represents the viscoelastic properties of the material [35]. Thus, a larger loss factor suggests a more viscous material and a smaller loss factor suggests a more elastic material. Within a certain range, it can indicate the state of the material and reflect the size of the moist area.

#### 3.2.4 Cryo-SEM.

We used cryo-SEM to observe the structure of the gel patch and preserve the original structure of the hydrogel. The sample was first frozen to the temperature of liquid nitrogen. It was fixed at a low temperature, the solid water was vaporized in a vacuum environment, and finally, the structure was observed using cryo-SEM [36–38].

#### 3.2.5 TGA-MS.

TGA is often used to study heating-induced dissociation, dehydration, decomposition, and oxidation/reduction of substances by measuring temperature-related changes in mass. The resulting thermogram shows distinguished mass loss steps that correspond to the thermal decomposition stages of different components [39], and their contents can be directly read based on the thermogram [40,41]. To gain a deeper understanding of the degradation process, the outgassing during heating was analyzed using MS. Four PNSPs were measured to investigate whether the weight loss curves of material with the same degree of neutralization (AH-105X and NP-700, AH-106X, and NP-800) were the same by TGA. Thereafter, we investigated whether the weight loss curves of four gel patches with the same degree of neutralization (GP-AH-105X and GP-NP-700, GP-AH-106X and GP-NP-800) were the same, determining whether the chemical properties of the materials had changed. The weight loss curves of four gel patches with the same degree of neutralization (neutrality level 50% and neutrality level 35%) were observed to detect any changes in weight loss curves and the properties of PNSPs. The temperature increased from 25 °C to 500 °C, with a warming speed of 20 °C/min，The nitrogen gas flow rate is 60 mL/min，Crucible type is platinum(100 μL).

#### 3.2.6 Statistical analysis.

Statistical analysis was performed using GraphPad Prism 10.12 software. Experimental data are expressed as mean ± standard (mean ± SD). Intergroup comparisons were analyzed using one-way ANOVA. P < 0.05 indicates a statistically significant difference.

## 4. Results

### 4.1 The results of the Chinese pharmacopeia item test

#### 4.1.1 Formability results.

**Fig 3:**
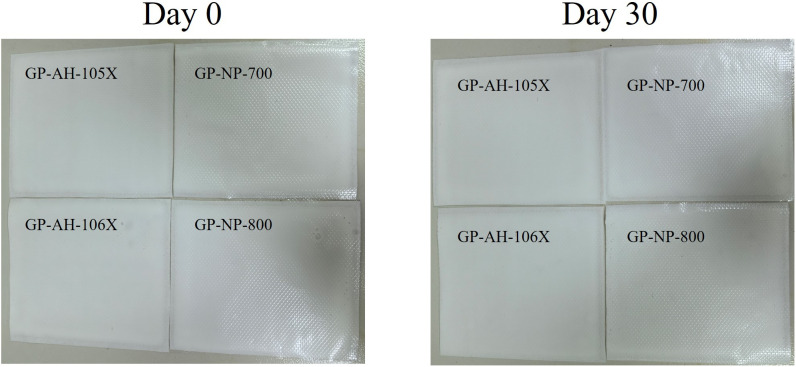
Day 0 and Day 30 gel patches.

The four gel patches were measured on days 0 and 30 after gel patch formation (Fig 3). The surface of four gel patches (GP-AH-105X, GP-NP-700, GP-AH-106X and GP-NP-800) were free of flow and in compliance with the regulations. Polyacrylic acids with the same neutralization degree do not affect the formation of the gel patch.

#### 4.1.2 Adhesiveness results.

After the maturation of the gel patch preparation, the PNSPs and initial adhesion of the gel patch were evaluated to ensure its quality and compliance in subsequent tests.

At days 0 and 30 after gel patch formation, the maximum ball numbers that could be stuck were 25 and 25 for GP-AH-105X and 25 and 24 for GP-NP-700, respectively (Table 2). The maximum ball numbers that could be stuck were 29 and 27 for GP-AH-106X and 29 and 26 for GP-NP-800, respectively. The adhesiveness of gel patches GP-AH-105X and GP-NP-700 exhibited almost no difference on days 0 and 30, and the adhesiveness of GP-AH-106X and GP-NP-800 showed a slight decrease on day 30 compared to day 0. The overall adhesiveness of GP-AH-106X and GP-NP-800 (35% neutralization) was greater than that of GP-AH-105X and GP-NP-700 (50% neutralization). The acrylic acid content in partially neutralized polyacrylic acid has a certain influence on the tackiness of gel patches [3].

**Table 2:** Adhesiveness test results (n = 3).

Gel patch	Day	Angle of inclination	Ball number
GP-AH-105X	Day 0	30°	25
	Day 30		25
GP-NP-700	Day 0		25
	Day 30		24
GP-AH-106X	Day 0		29
	Day 30		27
GP-NP-800	Day 0		29
	Day 30		26

#### 4.1.3 Peel strength results.

**Fig 4:**
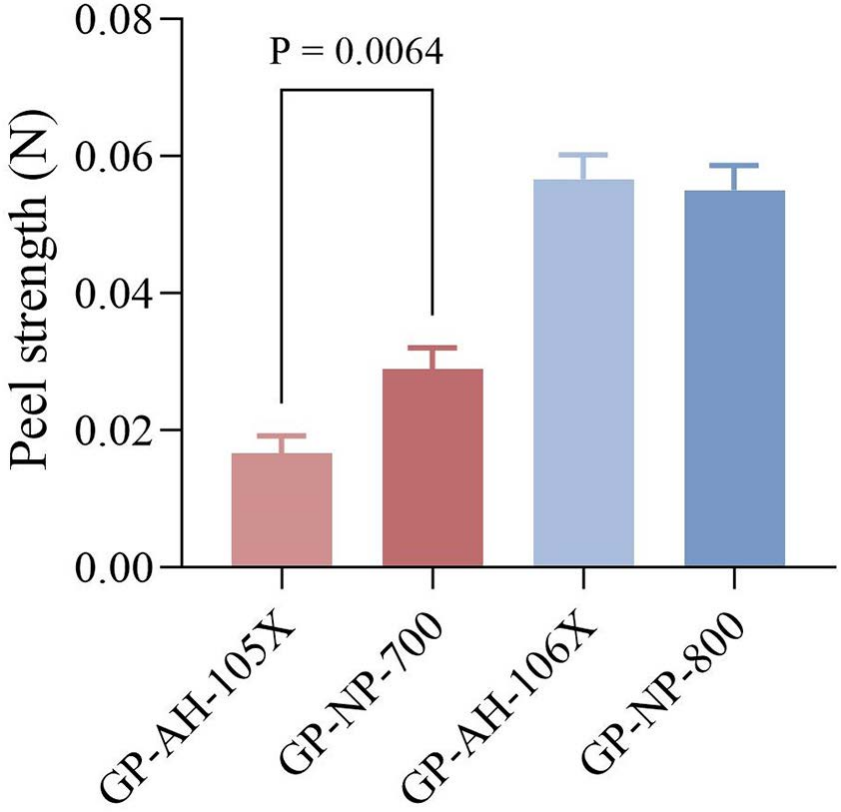
Peel strength results of the gel patches (n = 3).

The peel strength of four gel patches was examined, with the following order of magnitude: GP-NP-700 > GP-AH-105X and there is no significant difference between GP-AH-106X and GP-NP-800. (Fig 4). This indicates that even when the degree of neutralization of the skeleton material is identical, the peel strength of the gel patches may not necessarily be the same.

### 4.2 Electronic nose test

**Fig 5:**
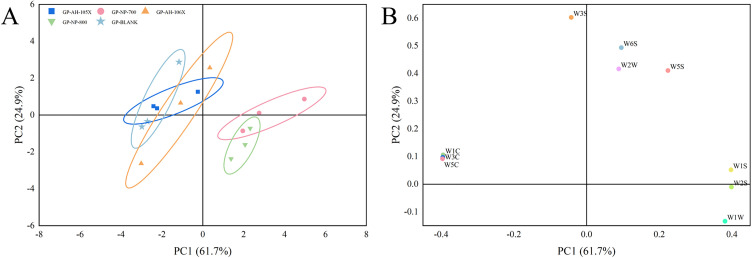
A: PCA of four gel patches (n  =  3). B: Loading plot of four gel patches (n  =  3).

PCA is the data transformation and dimensionality reduction of the extracted sensor response signals and the linear classification of the reduced feature vectors. The variance contribution ratio of the 1st principal component and the 2nd component obtained in the PCA transformation were included in PC1 and PC2. With a larger variance contribution ratio, this principal component can better reflect the information of the original multiple indicators. The results of PCA analysis of the four gel patch samples are shown in Fig 5A. In the correlation matrix mode, the distinguishing contribution rate of the PC1 was 61.7% and the distinguishing contribution rate of the PC2 was 24.9%, indicating that the two-dimensional PCA plot can fully characterize all information from the original data of the four gel patches. The odors of GP-AH-105, GP-AH-106X, and the blank matrix are relatively similar. The odors of GP-NP-700 and GP-NP-800 are relatively similar and completely distinct from the blank matrix. As shown in Fig 5B and Table 3, the sensors contributing most significantly to PC1 are W1S and W2S. In PC2, the sensor contributing most significantly is W3S.

**Table 3:** Sensitive substances for electronic nose sensors.

Sensor number	Sensor code	Sensitive substances
1	W1C	Aromatic hydrocarbons
2	W5S	Nitrogen oxides
3	W3C	Ammonia, aromatic molecules
4	W6S	hydride
5	W5C	Olefins, aromatics, polar molecules
6	W1S	alkanes
7	W1W	sulfur compounds
8	W2S	Alcohols, certain aromatic compounds
9	W2W	Aromatic hydrocarbons, organic sulfur compounds
10	W3S	Alkanes and aliphatic compounds

An interesting phenomenon was observed in the PCA two-dimensional diagram of the electronic nose: gel patches made from PNSP with a neutralization degree of 50% exhibited distinct odors, as did those made from gel patches with a neutralization degree of 35%. Conversely, gel patches produced from PNSP within the same series exhibited similar odors. This may overturn our everyday assumption that materials with the same neutrality must possess identical properties. We speculate that acrylic acid monomer is the primary raw material for PNSP. During production, even minor differences in the initiators and their decomposition products used in the polyacrylic acid polymerization reaction, as well as slight variations in polymerization temperature, reaction pressure, and duration, can all exert some influence on the final product. Furthermore, even when raw materials possess identical neutralization levels, factors such as particle morphology and crosslinking degree can influence their swelling and dispersion behavior within the matrix. This, in turn, alters the release kinetics of volatile components, potentially resulting in varying odor intensities on the patch surface. Therefore, the effect of PNSP on the odor of gel patches cannot be attributed solely to its neutralization degree. Multiple factors, including its synthetic raw materials and production process, should be considered.

### 4.3 Rheology results

The gel patch is a semi-solid formulation, and rheological analysis can provide objective data on its internal structure, viscosity, and spreadability [42]. The rheological properties of the matrix material are a key factor influencing the rheological behavior of semi-solid formulations such as gel patches. In-depth research on these properties can provide guidance for formulation optimization and establish a scientific foundation for the quality consistency evaluation of gel patches.

#### 4.3.1 Amplitude scanning.

**Fig 6:**
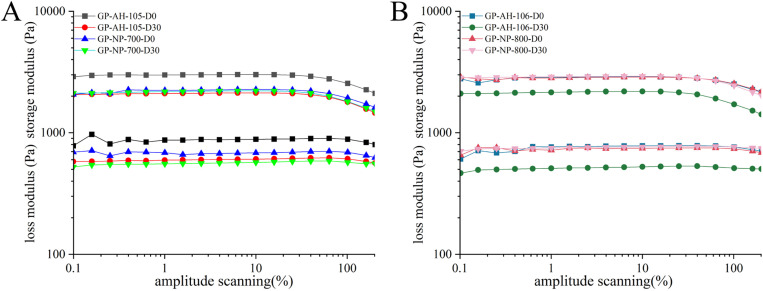
A: Amplitude scanning of GP-AH-105X and GP-NP-700; B: Amplitude scanning of GP-AH-106X and GP-NP-800.

Amplitude scans were performed on the four gel patches at days 0 and 30 after formation. Results showed (Fig 6) that the linear viscoelastic range of gel patches prepared from PNSP with identical neutralization degrees was essentially consistent. This indicates that despite differing source materials for the backbone, maintaining the same neutralization degree ensures consistent stability within the gel patch systems.

#### 4.3.2 Frequency sweep and loss angle tangent.

**Fig 7:**
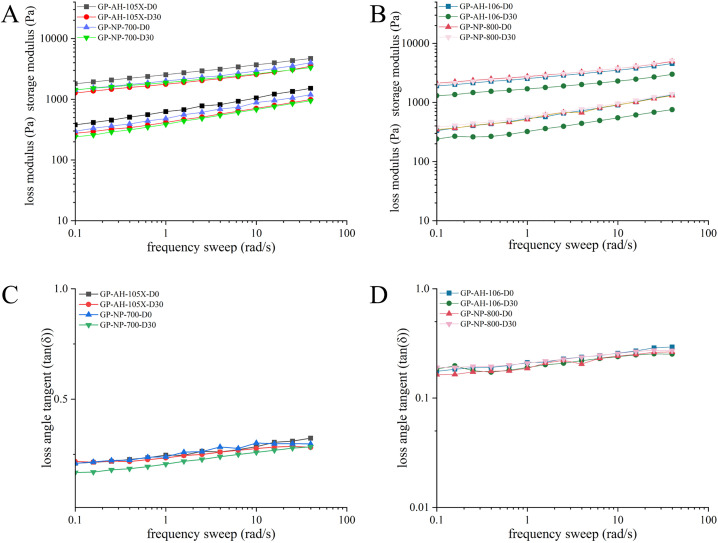
A: Frequency sweep of GP-AH-105X and GP-NP-700; B: Frequency sweep of GP-AH-106X and GP-NP-800; C: Loss angle tangent of the preparation of GP-AH-105X and GP-NP-700; D: Loss angle tangent of the preparation of GP-AH-106X and GP-NP-800.

The viscoelastic properties of the gel patches were measured on day 0 and day 30 after formation. Figs 7A and 7B shows the viscoelastic curve of a gel patch prepared from partially neutralized sodium polyacrylate with 35% neutralization degree. On day 0, G′ (GP-AH-105X) > G′ (GP-NP-700), the G′ value of GP-AH-106X was nearly identical to that of GP-NP-800. By Day 30, the G′ value of GP-AH-105X was nearly identical to that of GP-NP-700, G′ (GP-AH-106X) < G′ (GP-NP-800). This indicates that G′ does undergo changes, but the extent of these changes is relatively small.

From the preceding discussion, we know that loss angle tangent is the ratio of G″ to G′. As shown in Fig 7C and 7D, within 30 days, the loss factors of all four gel patches remained below 1, indicating that G′ exceeded G″. This suggests a viscoelastic behavior leaning more toward elasticity, with a robust structure that resists flow, exhibiting solid-like characteristics. Furthermore, the loss factors showed no significant changes over the 30-day period, indicating that the internal structure of the framework material remained stable after forming a network structure, with no noticeable alterations even under long-term storage conditions.

Preparing gel patches using the same formulation and sodium polyacrylate with identical neutralization levels implies that the degree of ionization and charge density of the polyacrylic acid chains in the samples are consistent. Consequently, the rheological characteristics of the samples should be identical or similar. However, based on the rheological data presented above, gel patches prepared using PNSP with neutralization degrees of 35% and 50% exhibit slight differences in their G′ values. This indicates that even with identical neutralization degrees, the rheological properties of the resulting gel patches may still vary to some extent.

### 4.4 The results of structural analysis

**Fig 8:**
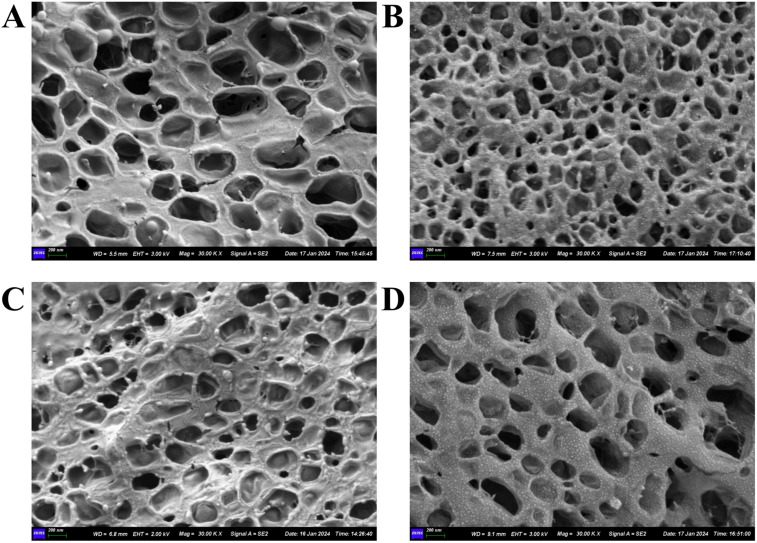
A: GP-AH-105X, bar: 200 nm; B: GP-NP-700, bar: 200 nm; C: GP-AH-106X, bar: 200 nm; D: GP-NP-800, bar: 200 nm.

**Fig 9:**
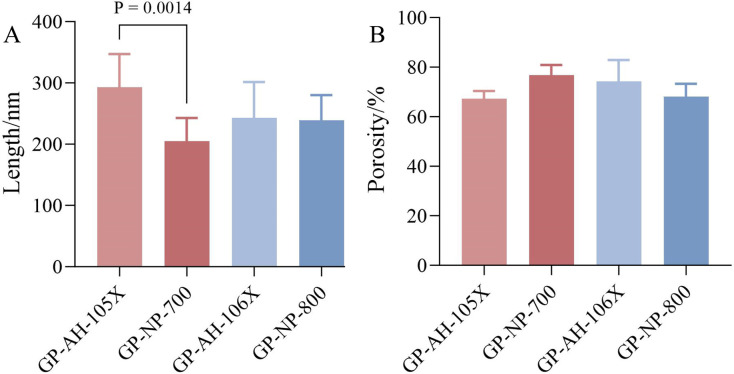
A: The size of the voids in the three-dimensional network structure of gel patches (n  =  10); B: Percentage of pore volume in the 3D Structure of gel patches (n  =  3).

Structural observations of the four gel patches on Day 0 after formulation were conducted, as shown in Fig 8A–8D. All four gel patches exhibited a 3D network structure, indicating that the cross-linking of the scaffold material was complete and the formulation preparation was successful. Fig 9A and 9B show the pore size and the porosity ratio of the 3D network structure, respectively. Based on the size of the voids in the 3D network structure and the proportion of porosity, it can be determined that GP-AH-105X exhibits large pores with a low void fraction, forming a macroporous, sparse 3D network structure, whereas GP-NP-700 features small pores with a high void fraction, constituting a microporous, dense 3D network structure. The GP-AH-106X features small pores with a high void ratio, forming a dense 3D mesh structure. Conversely, the GP-NP-800 exhibits large pores with a low void ratio, creating a sparse 3D mesh structure. Rheological data from the Day 0 frequency scan indicates that the G′ value of GP-AH-105X is higher than that of GP-NP-700, while the G′ values of GP-AH-106X and GP-NP-800 are relatively close. Initially, we hypothesized that the internal 3D network structure within gel patches would influence their rheological properties in semi-solid formulations. For instance, a large-pore, sparse 3D network structure would exhibit a lower G′ than a small-pore, dense 3D network structure. However, based on the 3D network structures and rheological results of GP-AH-105X and GP-AH-106X, no direct correlation was observed between the two.

### 4.5 The results of TGA-MS

**Fig 10:**
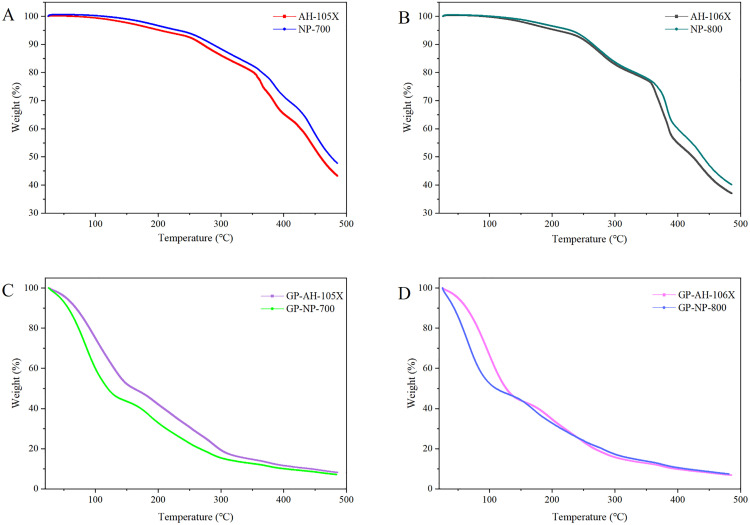
A: Thermogravimetric analysis diagram of AH-105X and NP-700; B: Thermogravimetric analysis diagram of AH-106X and NP-800; C: Thermogravimetric analysis diagram of GP-AH-105X and GP-NP-700; D: Thermogravimetric analysis diagram of GP-AH-106X and GP-NP-800.

For PNSPs, when the temperature ranged from room temperature to 250 °C, the thermogravimetric differential curve remained relatively stable with no significant weight loss (Fig 10A and 10B). Between 250 °C and 500 °C, the weightlessness curve indicated that the weight of ingredients decreased with the increase in temperature, with a decelerating rate. This finding suggests that PNSPs is decomposing and vaporizing at high temperatures. The heat loss curves of PNSPs with the same degree of neutralization were almost identical.

**Fig 11:**
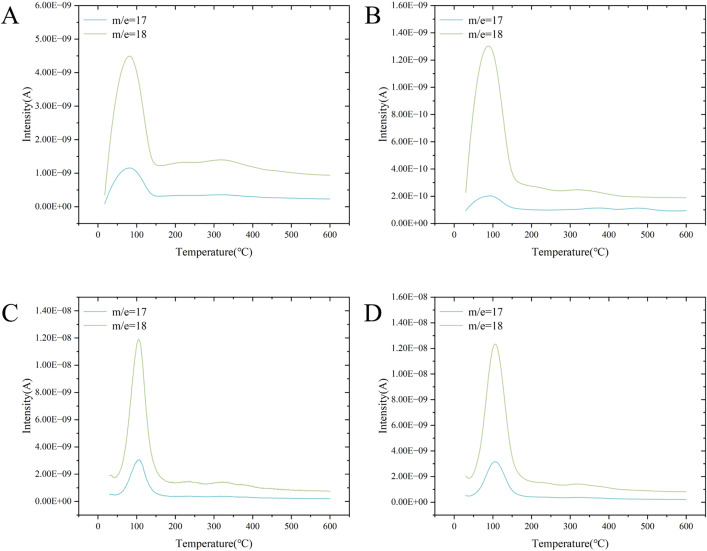
A: Mass to charge ratio of GP-AH-105X; B: Mass to charge ratio of GP-NP-700；C: Mass to charge ratio of GP-AH-106X; D: Mass to charge ratio of GP-NP-800.

For the gel patches, the weightlessness curve indicated a dramatic weight loss of the preparation sample around 100 °C (Fig 10C and 10D), indicating that a large amount of water in the sample was vaporized and the vaporization rate remained relatively fast. MS coupling provides molecular-level verification. As shown in Fig 11A–11D, m/e = 18 corresponds to water molecules (H₂O⁺), the primary characteristic ion of water, while m/e = 17 likely originates from water fragment ions (OH⁺). Therefore, mass loss at this temperature can be attributed to water evaporation. As the temperature increased from 150 °C to 300 °C, the weightlessness curve showed that different substances in the preparation were decomposing and vaporizing, with a fairly gentle rate of weight loss. The weightlessness curve tended to stabilize between 300 °C and 500 °C. Although the weight loss curve profiles of GP-AH-105X and GP-NP-700 were generally the same, the ratio column of their weight loss at the same temperature was different (Fig 10C). The weight loss curves and ratios of GP-AH-106X and GP-NP-800 were almost the same at 100–500 °C, although there were some differences in the weight loss rate at temperatures less than 100 °C (Fig 10D).

Through the above studies, we observe that PNSP with identical neutralization levels exhibits consistent thermal stability. When used as a gel matrix material to form gel patches, the formulations also display similar thermal behavior. This suggests consistency in the internal structure and interactions within the formulations, while also indicating that incorporating matrix materials from different sources does not alter the stability of the formulations.

## 5. Discussion and conclusion

The electronic nose is an intelligent analytical device capable of simulating human olfaction. Compared to traditional human sensory evaluation, it can effectively mitigates the influence of evaluators’ psychological and physiological states, significantly reducing evaluation errors [43]. The odor of pharmaceutical formulations is as important as their appearance and taste. Unpleasant drug odors can easily trigger patients’ resistance, while pleasant odors can help improve patients’ medication experience. Therefore, we introduce an electronic nose to evaluate the odor of gel patch. The experimental data provided by electronic nose is not only more scientific and reliable but can also be combined with data modeling for the visualization.

**Fig 12:**
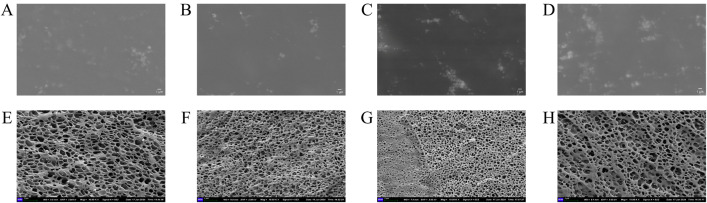
A: GP-AH-105X, bar: 1 μm; B: GP-NP-700, bar: 1 μm; C: GP-AH-106X, bar: 1 μm; D: GP-NP-800, bar: 1 μm; E: GP-AH-105X, bar: 1 μm; F: GP-NP-700, bar: 1 μm; G: GP-AH-106X, bar: 1 μm; H: GP-NP-800, bar: 1 μm.

We evaluated the structure of the gel patch to confirm the complete evaporation of water, determine the temperature and humidity during the test and its vacuum level, and assess the internal structure of the gel patch. In the experimental process, a vacuum drying oven was used to remove the moisture in the gel patch. At the same time, we ensured that external moisture could not enter into the vacuum-drying oven. Due to the limited vacuum of the vacuum drying box, it was difficult to control the temperature and humidity of the outside world when removing the sample; therefore, the internal structure of the gel patch could not be observed. Secondly, the researchers used the freezing method of gradual cooling to condense the moisture in the gel patch into ice. Then, sublimation was used to remove the moisture inside the gel patch using the vacuum dryer. SEM was employed to observe the internal structure of the gel patch. It was found that freeze-drying could not remove the bound water of the gel patch; thus, SEM could not observe its internal structure (Fig 12A–12D). After many attempts, it was not possible to completely remove the water inside the gel patch to observe its structure. However, due to differences in the preparation methods of the gel patches, SEM can be used for structural characterization of some gel patches. Xingkai Ju [44] et al. studied ion-gel photothermal patch for skin tumor treatment and used SEM to characterize the structure of the gel patch to confirm that structure had achieved the expected regular array structure. SEM images clearly demonstrated that the microgel exhibited a porous structure in a dehydrated state, directly confirming the success of the preparation process and laying the foundation for subsequent gradient drug loading and in vivo experiments. In this experiment, the gel patch exhibited a paste-like consistency, requiring strict conditions for freeze-drying. Therefore, an integrated cryo-SEM was used to observe the structure of the gel patch (Fig 12E–12H). Cryo-SEM fills the gap of traditional SEM in the characterization of water-containing, soft matter, and biological samples through cryo-sampling and cryo-imaging technology. It can become an indispensable tool in the fields of materials [45], biology [46], and medicine [47]. Based on the experimental data from SEM in the literature, it can be proven that SEM can observe the structure of formulations. However, in this study, Cryo-SEM is superior to SEM for formulation with high water content and strict detection conditions.

TGA is widely employed to determine the changes in the heat loss of materials. Gunjan Sarkar [48] et al. used TGA to assess the thermal stability of materials used in the preparation of transdermal patch. According to the TGA experimental data, the temperature range of 40–110 °C corresponds primarily to water evaporation, while the temperature range of 230–310 °C corresponds to the decomposition of polymer materials. The maximum decomposition rate temperature of 281 °C and the half-weight loss temperature of 293 °C are both higher than those reported in the literature for okra mucilage. The TGA experimental data indicate that taro corms mucilage has superior stability, indirectly suggesting that taro corms mucilage has certain processing feasibility as a material for transdermal patches. In this experiment, we first used TGA to measure the changes in the mass of PNSP with the same degree of neutralization under heating conditions to investigate whether the composition of substances during sublimation is the same for PNSP with the same degree of neutralization. We also investigated whether the heat loss curves of PNSPs with the same degree of neutralization were the same after they were prepared into gel patches (GP-AH-105X and GP-NP-700, GP-AH-106X, and GP-NP-800). Furthermore, we investigated whether the properties of PNSPs changed after preparing them into gel patches to confirm that the properties of the excipients were stable. These findings can help determine the effect on drug release when PNSPs with the same degree of neutralization are incorporated into formulations. From our discussion, we can conclude that TGA can be used to analyze the stability of materials and provide certain data support for formulation research.

The experimental results above indicate that gel patches fabricated from matrix materials with identical neutralization levels exhibit distinct physical properties. These variations in physical characteristics may influence differences in transdermal drug release rates, local drug retention, and ultimately, in vivo pharmacodynamic effects. Therefore, based on this study, we will subsequently conduct in vitro release studies, transdermal experiments, and animal model validation to clarify the specific pharmacodynamic effects of partially neutralized sodium polyacrylate with equivalent neutralization levels. This will advance the standardized development and quality control of such patches.

Due to differences in the substitution of acrylic acid and sodium acrylate, PNSPs with different degrees of neutralization lead to differences in the physicochemical properties of the products, which in turn affects product properties and the quality of the gel patch as a matrix material. However, few studies have compared PNSPs with the same degree of neutralization but different models. Therefore, in the present study, two commonly used PNSPs with two degrees of neutralization were prepared into corresponding gel patches. These four corresponding gel patches were analyzed for their quality characteristics, odor properties, rheological properties and other attributes.

Comprehensive evaluations were conducted on days 0 and 30 after the formation of the gel patches. The results on the formability and adhesiveness of the gel patches indicate that partially neutralized sodium polyacrylate with incomplete neutralization does not affect the formability of the gel patches but does exert a certain influence on their adhesiveness. Electronic nose test results indicate that when analyzing the odor of gel patches, one cannot solely consider the neutrality of the matrix material; a comprehensive analysis from multiple perspectives is required. Rheological data from the four gel patches reveal that within the same LVR range, none of the patches exhibited deformation, indicating a relatively stable internal structure. Concurrently, G′ consistently exceeded G″, demonstrating solid-like characteristics. Cryo-SEM results clearly show that all four gel patches formed three-dimensional network structures, indicating successful preparation of the formulations. Thermogravimetric analysis indicates that neutralized polyacrylic acid with identical neutralization degrees exhibits comparable thermal stability. Incorporating backbone materials with the same neutralization degree but differing sources does not alter the formulation’s stability. Therefore, the degree of neutralization of gel matrix materials can be used to evaluate the physicochemical properties of formulations. However, reliance on neutralization alone is insufficient; similarities and differences between formulations require comprehensive examination from multiple perspectives.
